# Ischiocavernosus Muscle Release for Urethral Obstruction Treatment after Pelvic Symphyseal Distraction Osteotomy in a Cat with Pelvic Stenosis

**DOI:** 10.3390/vetsci8100225

**Published:** 2021-10-12

**Authors:** Yoon-Ho Roh, Jeong-Nam Kim, Pill-Moo Byun, Dae-Hyun Kim, Seong-Mok Jeong, Hae-Beom Lee

**Affiliations:** College of Veterinary Medicine, Chungnam National University, Daejeon 34134, Korea; 202020036@g.cnu.ac.kr (Y.-H.R.); papable@naver.com (J.-N.K.); mooche.byun@gmail.com (P.-M.B.); vet1982@cnu.ac.kr (D.-H.K.); jsmok@cnu.ac.kr (S.-M.J.)

**Keywords:** cat, pelvic stenosis, urethral obstruction, symphyseal distraction osteotomy

## Abstract

Symphyseal distraction osteotomy (SDO) with a polymethyl methacrylate (PMMA) spacer is an effective surgical treatment for cats with pelvic stenosis. This study reports the successful treatment of urethral obstruction due to ischiocavernosus muscle (IM) tension after SDO with a PMMA spacer. A 2-year-old castrated male Korean domestic shorthair feline had megacolon and pelvic canal stenosis. The ratio of the maximal diameter of the colon to the L5 length and the pelvic canal diameter ratio were 1.6 and 0.45, respectively. Pelvic SDO was performed with a PMMA spacer, leading to pelvic canal enlargement (pelvic canal diameter ratio: 0.73). Two days after surgery, dysuria was identified immediately after removing the preoperatively placed urinary catheter. Complete blood counts and serum biochemical profiles were within the reference intervals, and a positive contrast retrograde urethrogram confirmed urethral obstruction at the level of the membranous–pelvic urethra region. Increased tension of the IM leading to a narrowed urethra was suspected as the cause of urethral obstruction. After IM release at the level of origin on the ischium, the patient had an uneventful recovery with spontaneous urination. Muscle release resulted in excellent functional restoration, with no intraoperative or postoperative complications reported during the 12-month long-term follow-up. Therefore, SDO with IM release could be a feasible therapeutic option for severe pelvic stenosis without complications, such as urethral obstruction, in cats.

## 1. Introduction

Pelvic stenosis is the most common complication secondary to pelvic fractures, leading to dyschezia and severe gastrointestinal disease in cats [1]. A pelvic fracture or sacroiliac fracture or luxation can lead to medial displacement of the bone fragments and a narrow pelvic canal [2]. Other causes of pelvic stenosis include congenital dysplasia of the vertebrae and metabolic disorders. Conservative management with strict cage rest, diet, and stool softeners, such as laxatives, can be used for functional restoration. Nevertheless, surgical treatment would be needed when cats experience discomfort due to medially displaced bone fragments and complications, including dystocia in queens and rectal impingement with chronic obstipation [2,3,4]. Moreover, early surgical treatment is needed in cats to prevent irreversible megacolon within 6 months of the initial trauma [5,6].

There are several reports of surgical treatment involving pelvic widening techniques, including triple pelvic osteotomy, partial pelvectomy, redirecting the ilium, and symphyseal distraction osteotomy (SDO) with or without subtotal colectomy to reduce clinical signs [2,4,7,8,9]. All treatments resulted in favorable outcomes [2,10]. SDO is a common technique because it is simple to perform and can help adjust the pelvic canal using a spacer. Moreover, it does not require a substantial amount of soft tissue dissection, leading to sciatic and obturator nerve and urethra or rectum injury, compared with hemipelvectomy and triple pelvic osteotomy. Various types of spacers, including metal spacers, bone spacers, and polymethyl methacrylate (PMMA), can be used for the stable configuration of the hemipelvis after symphyseal osteotomy [4]. However, this technique could lead to several postoperative complications, such as worsening constipation due to unfavorable distraction, dislodgement of the spacer, and additional pelvic bone fractures due to postoperative increased tension and inflammation following implantation [2,3,4,8,10].

Although a few postoperative complications after SDO with spacers in cats with pelvic stenosis have been reported, there are no reports regarding secondary urethral obstruction due to IM tension after SDO with a PMMA spacer [3,4,11]. Furthermore, to the best of our knowledge, there are no reports regarding secondary urethral obstruction due to IM tension after SDO with a PMMA spacer. This report describes the clinical features, treatment, and outcomes of a feline manifesting postoperative urethral obstruction following increased tension of the ischiocavernosus muscle (IM) after SDO with a PMMA spacer.

## 2. Case Description

### 2.1. Case

**Figure 1:**
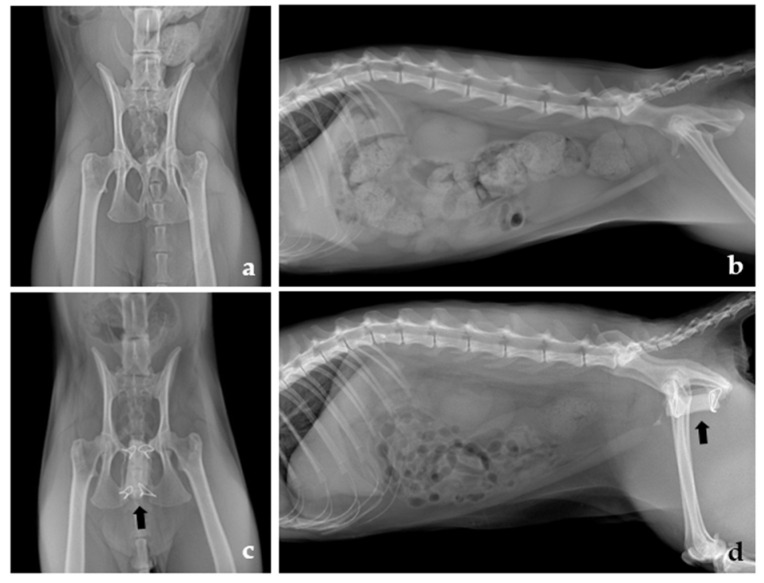
Preoperative ventrodorsal and lateral radiographs of the pelvis (**a**,**b**). There is colonic distention with feces and pelvic canal stenosis. Postoperative ventrodorsal and lateral radiographs of the pelvis (**c**,**d**) showing a polymethyl methacrylate spacer (black arrow) placed in the symphyseal osteotomy and the distracted pelvis 40 days after surgery with no colonic distention.

A 2-year-old castrated male Korean domestic shorthair feline was presented for obstipation investigation. According to the owner, the feline had a traumatic insult before adoption. After adoption (1 year old at that time), he was managed with medical treatment including lactulose and cisapride for 1 year at the local hospital. However, he had a good appetite and no lameness. Rectal examination revealed pelvic narrowing on the insertion of fingers and significant pain and compression reaction. Additionally, radiographic examination revealed colonic distentions with feces, asymmetrical pelvic bone alignment due to malunion of the fracture, and a reduced pelvic canal diameter (Figure 1a,b). The ratio of the maximal diameter of the colon was 1.6 times the length of the L5 [12]. These findings were suggestive of pelvic narrowing, as the pelvic canal diameter ratio was 0.45 [8,13]. Preanesthetic laboratory investigations comprising a complete blood cell count, serum biochemistry profile, and electrolytes were unremarkable, except for increased albumin (4.5 g/dL) (reverence interval (RI): 1.9–3.9) and amylase (1906 U/L) (RI: 550–1800) levels. Computed tomography (CT) (AlexionTM, Canon Medical Systems Corporation, Otawara, Japan) images of the pelvis were obtained in ventral recumbency with a slice thickness of 1 mm and operating parameters of 120 kilovolt (kV) and 12 milliampere (mA). CT also revealed an asymmetric pelvic bone due to malunion after trauma, leading to severe narrowing of the pelvic canal with a distended colon. Based on these examinations, pelvic SDO using a PMMA spacer was planned to increase the pelvic canal.

### 2.2. Surgical Technique

**Figure 2:**
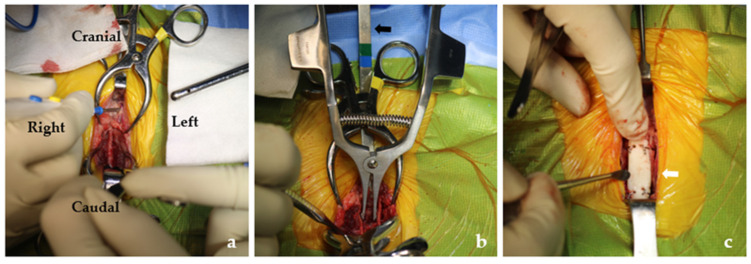
Ventral midline approach with elevation of the gracilis and adductor muscles (**a**). Distraction of symphysis is maintained with a distractor and spatula (black arrow) to avoid damaging the organ under the bone (**b**). A polymethyl methacrylate spacer (white arrow) was inserted and secured with a stainless steel wire (**c**).

The surgical technique used in the present study was similar to that used in previous studies [1,2]. Before surgery, an indwelling urinary catheter was inserted. General anesthesia was induced prior to surgery. The patient was stabilized with fluid therapy and underwent premedication with 0.1 mg/kg intravenous (IV) hydromorphone (Hana Pharm, Seongnam, Korea) and 0.2 mg/kg IV midazolam (Bukwang Pharmaceutical Co., Seoul, Korea). Anesthesia was induced with 4 mg/kg IV propofol (Hana Pharm, Seongnam, Korea) and maintained with 2% isoflurane (Hana Pharm, Seongnam, Korea). For prophylaxis, 22 mg/kg IV cefazolin (Chong Kun Dang Healthcare, Seoul, Korea) was administered at 90-min intervals throughout the surgery [14]. Dorsal recumbency with an abducted pelvic limb was performed. A standard ventral midline approach was used for the pelvis. The gracilis and adductor muscles were minimally elevated (Figure 2a). Four holes were made in the pubis and ischium using a 1.2 drill bit at either side of the symphysis as described in a previous report [2]. Subsequently, pelvic symphyseal osteotomy was performed, while the spatula was underneath the symphysis to protect the pelvic organs and muscles. Compression in the pelvic canal was examined while inserting an index finger, and nonoperative assistance into the rectum during the split bone was maintained with a distractor (Figure 2b). A PMMA prosthesis was crafted based on the size of the paper that was previously molded for the shape of the distracting site. Four holes were also made for the stainless steel wire (0.6 mm) into the PMMA spacer in the position of the hole in the pelvic bone (Figure 2c). The PMMA spacer was positioned in the gap between the split segments and secured through holes with stainless steel wires (Figure 2). The pressure alleviation was confirmed by finger insertion. The ventral midline was apposed and sutured routinely. Immediate radiographic examination postoperatively showed pelvic canal enlargement of 30% as the pelvic canal diameter ratio was 0.73 (Figure 1c,d).

### 2.3. Complication and IM Release

**Figure 3:**
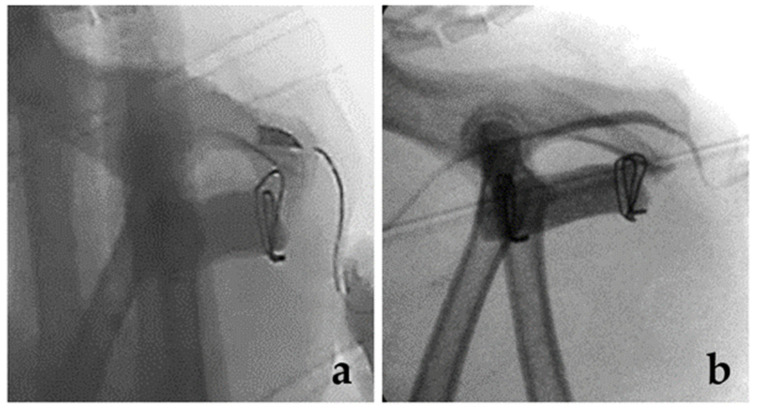
Postoperative retrograde urethrogram under the guide of C-arm fluoroscopy. Insertion of the urine catheter was impeded, and the narrow urethra was confirmed on the ischium level (**a**). Marked improvement of the contrast flow into the bladder was noted after ischiocavernosus muscle release (**b**).

**Figure 4:**
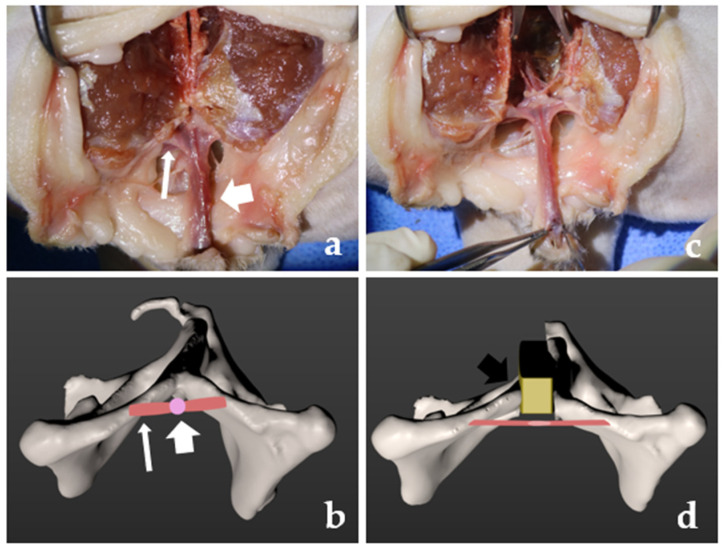
The ex vivo images (**a**,**c**) and schematization (**b**,**d**) of development of the urethra obstruction due to increased tension of ischiocavernosus muscle (white narrow arrow). The urethra (white broad arrow) could be stretched out and obstructed by distraction with an instrument or spacer (black arrow).

The patient recovered with no complications related to anesthesia and was managed with cefazolin (22 mg/kg, three times per day (TID) for 3 days, IV) and lactulose (JW Pharmaceutical, Seoul, Korea, 2–3 mL, TID for 3 days, per os (PO)). Urine production was closely monitored using a preoperatively placed indwelling urinary catheter. Normal defecation was observed on postoperative day (POD) 2. After removing the urinary catheter, dysuria was observed for 24 h on POD 2–3. Biochemistry revealed increased creatinine (5.1 (RI: 1–2) mg/dL), blood urea nitrogen (80.3 (RI: 18–33) mg/dL), glucose (306 (RI: 73–134) mg/dL), and aspartate aminotransferase (88 (RI: 12–46) U/L) levels, consistent with obstructive post-renal azotemia on POD 4. Abdominal radiography revealed marked bladder distention. Abdominal ultrasonography demonstrated no physiologic obstruction of the urethra. Replacement of a 3.5-Fr indwelling urinary catheter failed due to blockage of the urethra. A positive contrast retrograde urethrogram confirmed pelvic urethral obstruction at the level of the membranous–pelvic urethra region when inserting a 3.5-Fr urinary catheter (Figure 3a) [14]. Based on the clinical signs, history, and diagnostic imaging, urethral obstruction was suspected to be due to increased tension of the IM at the level of the membranous–pelvic urethra after symphyseal distraction (Figure 4). General anesthesia was induced. A ventral recumbency on the towel with an abducted pelvic limb was performed. Dorsal approaches to the ischium were performed on both sides. The IM was released near the ischium. Subsequently, urethral catheterization using a 3.5-Fr urinary catheter and retrograde urethrogram was performed, which confirmed the distended urethra (Figure 3b). Mild dribbling of urine was noted but resolved within 48 h with diazepam and prazosin. No urinary incontinence was observed. The patient recovered uneventfully after catheter removal on POD 3 during hospitalization. The cat was discharged with only a small dose of lactulose. Complication and recurrence of obstipation and urinary incontinence did not occur anymore until the final 12-month follow-up, although the patient was constantly managed with small doses of lactulose.

## 3. Discussion

We reported a case of urethral obstruction due to increased tension of the IM after symphyseal distraction and successful treatment using muscle release. SDO resulted in immediate favorable pelvic distraction (pelvic canal diameter ratio: 0.73). However, immediate postoperative complications, including stranguria and dysuria, were confirmed after removal of the indwelling urinary catheter placed before surgery. The urethral obstruction was caused by the narrowed urethra due to increased tension of the IM around the urethra. It was hypothesized that the excessive distracted pelvic bone led to increased tension of the muscle around the urethra in the pelvis. After IM release, the patient restored normal urine flow and had favorable conditions without any complications.

Urethral obstruction in cats is a common and life-threatening emergency, accounting for 9.0% of feline emergent cases [15,16]. The common causes of urethral obstruction include urethral plugs, urolithiasis, neoplasia, bladder displacement or herniation, and urethral structure and functional obstruction. The most common causes of urinary obstruction in cats are urethral plugs and uroliths. Additionally, urethral obstruction occurs more commonly in male cats due to their comparably long and narrow urethras with urethral plugs, uroliths, and idiopathic causes [16]. In our case, the narrow postoperative morphology of the urethra suggested the effect of increased tension of the muscle surrounding the urethra, specifically the IM. In our viewpoint, the urethral obstruction in this feline could be attributed to either (1) increased IM tension, (2) inflammation and edema surrounding the IM due to the surgical approach, (3) stress-induced abnormal function, or (4) unknown etiology.

Although a pathologic diagnosis could not be established because of the consequent lack of histopathology and the favorable outcome, immediate restoration of urine flow after IM release is compatible with a problem outside the urethra. Options (1) and (2) describe a scenario of urethral compression due to pelvic canal widening. This patient was accustomed to the narrow pelvic canal, and fibrotic tissue with muscle contracture could occur after a trauma accident. It is unlikely that the urethra is pressurized by mass effects outside the urethra. Inflammation or fibrotic tissue may not be overproduced, considering that the clinical symptoms occurred after removing the indwelling urinary catheter 3 days after surgery. Moreover, there was a minimally invasive approach to acquire sites for PMMA with no soft tissue dissection around the ischial arch during surgery. There was no inflammation with urolithiasis or other remarkable signs on the radiography or ultrasound images. Therefore, urethral stricture cannot be the cause of dysuria in this patient [17]. We tried to stabilize the condition without excessive bandage and catheterization in the urethra 2 days after surgery, because increased recurrence of urethral obstruction associated with long-term indwelling urethral catheterization has been reported [18]. Based on the anatomic features of the muscle and the bone surrounding the urethra, increased tension due to distraction of the pelvis might have compressed the urethra and compromised urine flow. The confirmation of urine flow on a retrograde urethrogram under the guidance of fluoroscopy after immediate muscle release could be supportive of the abovementioned clinical suspicion. However, there have been no reports of similar complication signs, although previous reports have described the use of pelvic symphyseal distraction for pelvic stenosis [1,3,5,8,19]. Therefore, it could be hypothesized that the distracted distance of the pelvic bone is important to determine whether compression of the urethra occurs postoperatively. It was hoped that releasing tensioned muscle in the second surgery would allow some degree of urethral decompression and permit better flow of urine. Palliative stenting was not required for management. Options (3) and (4) describe the possible anesthetic and surgical complications. Idiopathic diseases, such as feline idiopathic cystitis and anxiety, are clinical signs of cats when they are stressed [20]. Clinical signs of stranguria and dysuria are compatible with urethral obstruction due to stress-related dysuria [16]. Moreover, there may be neuromuscular problems related to muscle compression associated with anxiety. Although these factors may have affected the results, functional urinary obstruction should be diagnosed based on clinical and historical signs with the exclusion of anatomical and neurological lesions [18]. But the physical obstruction was confirmed through retrograde urethrography in our case. An etiologic diagnosis could not be definitively established in this case because this was the only case and due to the lack of histopathology. In our case, the fluoroscopic imaging findings of obstruction at the level of the ischium and unremarkable signs of the lower urinary tract could be compatible with urethral obstruction due to increased tension of the IM or inflammation of the muscle surrounding the urethra. However, there was no mass effect due to inflammation around the muscle at the time of muscle release [21].

Surgical treatment of the pelvis in cats is challenging, although satisfactory outcomes have been reported with no major complications in pelvic canal stenosis [3,4]. The indications for surgery for pelvic canal stenosis have been previously described, but there is a lack of standards regarding clinical signs and the extent of distraction in patients with pelvic canal stenosis [3,8]. In general, the distraction distance can be determined preoperatively on CT and X-ray images [9]. However, there are several difficulties in distracting the bone due to muscle contracture and fibrosis during surgery [22]. One study described how malpositioned contracture of the sacroiliac joint and fibrotic tissue would hamper the distraction of the hemipelvis [7]. It is significantly difficult for patients who have not been treated after more than 10 days of trauma to perform corrective surgery without cutting bones [1,5]. Additionally, the size and material of the spacer for SDO have not yet been determined with regard to postoperative clinical signs [3,4,7,8,17]. Although compression was the criterion in this study for the distance of distraction by inserting a finger into the rectum within the pelvic canal, excessive distraction (pelvic canal ratio: 0.73) could increase the tension of the surrounding muscle. Therefore, the diameter of the pelvic canal and urethra should be monitored for injury from trauma after osteotomy of the symphysis or increased tension around the muscles [3,20]. Urethral obstruction should be considered a differential postoperative complication of SDO in cats with a history of trauma. Because of the lack of a large number of case reviews about pelvic canal stenosis related to clinical signs, further correlation analysis of the pelvic canal diameter ratio, clinical signs, and purposed distracted distance of pelvic osteotomy is required for future successful SDO without complications.

## 4. Conclusions

This report describes an unusual case of urethral obstruction after SDO without concurrent urolith and urethral plugs, adding information to the limited veterinary and human medicine. Urethra obstruction after SDO in cats should be considered a complication by a surgeon who would perform SDO with spacers. Therefore, it is also necessary to consider IM release to prevent urethral obstruction due to excessive distraction when performing SDO in cats with a history of trauma and fractures.

## References

[1] Matthiesen D.T., Scavelli T.D., Whitney W.O. (1991). Subtotal colectomy for the treatment of obstipation secondary to pelvic fracture malunion in cats. Vet. Surg..

[2] Ferguson J. (1996). Triple pelvic osteotomy for the treatment of pelvic canal stenosis in a cat. J. Small Anim. Pract..

[3] Oh K.S., Choi S.J., Kim N.S., Kim M.S., Lee K.C., Lee H.B. (2014). Pelvic symphyseal distraction osteotomy for constipation management secondary to pelvic stenosis. J. Vet. Clin..

[4] Prassinos N.N., Adamama-Moraitou K.K., Gouletsou P.G., Rallis T.S. (2007). Symphyseal distraction-osteotomy using a novel spacer of spirally fashioned orthopaedic wire for the management of obstipation. J. Feline Med. Surg..

[5] Schrader S. (1992). Pelvic osteotomy as a treatment for obstipation in cats with acquired stenosis of the pelvic canal: Six cases (1978–1989). J. Am. Vet. Med. Assoc..

[6] Robert J.W., David H. (1999). Pathogenesis, diagnosis, and therapy of feline idiopathic megacolon. Vet. Clin. N. Am. Small Anim. Pract..

[7] Averill S., Johnson A., Schaeffer D. (1997). Risk factors associated with development of pelvic canal stenosis secondary to sacroiliac separation: 84 cases (1985–1995). J. Am. Vet. Med. Assoc..

[8] Cinti F., Cavaliere L., Degna M.T., Rossi F., Pisani G. (2020). Triple pelvic osteotomy fixed with lag screw for the treatment of pelvic canal stenosis in five cats. Vet. Comp. Orthop. Traumatol..

[9] Hamilton M.H., Evans D.A., Langley-Hobbs S.J. (2009). Feline ilial fractures: Assessment of screw loosening and pelvic canal narrowing after lateral plating. Vet. Surg..

[10] Yoon H.Y., Kim K.H., Jeong S.W. (2013). Hemipelvectomy in a cat with obstipation. J. Vet. Clin..

[11] Atallah F.A., Silva R.S., Oliveira A.L.D.A., Souza H.J.M. (2016). Subcolectomy and symphyseal distraction-osteotomy using a spacer of spirally fashioned orthopedic wire: A treatment option for cats with pelvic canal stenosis, megacolon and obstipation. Ciênc. Rural.

[12] Trevail T., Gunn-moore D., Carrera I., Courcier E., Sullivan M. (2011). Radiographic diameter of the colon in normal and constipated cats and in cats with megacolon. Vet. Radiol. Ultrasound.

[13] DeGroot W., Gibson T.W., Reynolds D., Murphy K.A. (2016). Internal hemipelvectomy for treatment of obstipation secondary to pelvic malunion in 3 cats. Can. Vet. J..

[14] Cuddy L.C., McAlinden A.B., Tobias K.M., Johnston S.A. (2018). Urethra. Veterinary Surgery: Small Animal.

[15] Goh C.S., Seim H. (2014). Feline perineal urethrostomy ventral approach. Today’s Vet. Pract..

[16] Segev G., Livne H., Ranen E., Lavy E. (2011). Urethral obstruction in cats: Predisposing factors, clinical, clinicopathological characteristics and prognosis. J. Feline Med. Surg..

[17] Currarino G. (1970). Narrowings of the male urethra caused by contractions or spasm of the bulbocavernosus muscle: Cysto-urethrographic observations. Am. J. Roentgenol. Radium Ther. Nucl. Med..

[18] Seitz M.A., Burkitt-Creedon J.M., Drobatz K.J. (2018). Evaluation for association between indwelling urethral catheter placement and risk of recurrent urethral obstruction in cats. J. Am. Vet. Med. Assoc..

[19] Bertoy R.W. (2002). Megacolon in the cat. Vet. Clin. N. Am. Small Anim. Pract..

[20] Forrester S.D., Towell T.L. (2015). Feline idiopathic cystitis. Vet. Clin. Small Anim. Pract..

[21] DeCamp C.E., Piermattei D., Flo G., Brinker W. (2016). Fracture of the Pelvis. Brinker, Piermattei and Flo’s Handbook of Small Animal Orthopedics and Fracture Repair.

[22] Witte P., Scott H. (2012). Conditions of the feline pelvic region. Pract..

